# An *in vivo* study of isotropic and anisotropic wall stress in a hyperelastic holzapfel-gasser-ogden model in the human abdominal aorta: Effects of age and sex

**DOI:** 10.3389/fphys.2023.1128131

**Published:** 2023-03-13

**Authors:** Jerker Karlsson, Jonas Stålhand, Carl-Johan Carlhäll, Toste Länne, Jan Engvall

**Affiliations:** ^1^ Department of Clinical Physiology in Linköping, Department of Health, Medicine and Caring Sciences, Linköping University, Linköping, Sweden; ^2^ Solid Mechanics, Department of Management and Engineering, Linköping University, Linköping, Sweden; ^3^ Center for Medical Image Science and Visualization, Linköping University, Linköping, Sweden; ^4^ Department of Thoracic and Vascular Surgery in Linköping, Department of Health, Medicine and Caring Sciences, Linköping University, Linköping, Sweden

**Keywords:** abdominal aorta, remodeling, wall stress, sex, age

## Abstract

**Background:** Wall stress of the abdominal aorta (AA) appears to be an important factor in the assessment of risk for rupture based on the relationship between blood pressure and aortic diameter. We therefore investigated peak wall stress as well as isotropic and anisotropic wall stress of AA.

**Methods:** Thirty healthy adults (male = 15) were included. Pulsatile diameter changes were determined non-invasively by an echo-tracking system, and intra-aortic pressure was measured simultaneously. A computer based mechanical model was used to compute the isotropic and anisotropic components of circumferential and longitudinal stresses.

**Results:** Elderly males had higher total wall stress and a higher isotropic stress component in the circumferential direction and higher total longitudinal wall stress than elderly females. The isotropic component increased with age in males but not in females, whereas the anisotropic component decreased with age in both sexes.

**Conclusion:** We found that isotropic and anisotropic properties of the abdominal aortic wall differ between young and elderly participants and between the sexes. A possible explanation could relate to chemical alterations (e.g., due to sex hormones) and changes over time in the physical distribution of fibers. Modeling of wall stress components of the human AA may contribute to a better understanding of elastin-collagen interactions during remodeling of the aortic wall.

## What this paper adds

Stress is measured on a regular basis in the laboratory, i.e., *ex vivo*, *ex situ*. However, measuring stress *in vivo* is a difficult task. This paper presents quantitative estimates of circumferential and longitudinal stress for the human abdominal aorta stratified for age and sex, based on *in vivo* and *in situ* measured radius and pressure. Furthermore, estimates of the isotropic and anisotropic stress components are presented.

## Introduction

Several factors influence the formation, growth, and rupture of an abdominal aortic aneurysm (AAA). In humans, cellular mechanisms that operate in the abdominal aortic (AA) wall contribute to aneurysm formation through various tissue constituents such as elastin, collagen, and smooth muscle (Quintana and Taylor, 2019). Hypertension aggravates the risk of developing an AAA, and peak wall stress (PWS) has been implicated as a predictor of AAA rupture (Moll et al., 2011). Additionally, material anisotropy has been shown to influence the magnitude and distribution of PWS in AAAs in computer models (Rodriguez et al., 2008).

The ability to withstand hemodynamic forces, such as wall and shear stresses, will depend on the underlying architecture and constituents of the aortic wall. These forces, for which wall stress is much higher than shear stress, will induce either normal age-related remodeling or pathological processes, such as premature atherosclerosis or aneurysmal dilation. Notably, wall stress is considered important for aneurysm growth and rupture (Moll et al., 2011). Consequently, factors influencing the mechanical properties of a healthy aortic wall may also contribute to the understanding of the pathophysiologic conditions inducing AAA.

Earlier studies in healthy males and females have shown that wall stress in the AA is higher than wall stress in other large arteries and that males have higher wall stress than females (Åstrand et al., 2003). An age-related increase in arterial diameter with a compensatory thickening of the arterial wall, maintains a constant circumferential wall stress, except in male aortas in which stress increases with age (Astrand et al., 2005). In arterial mechanics, the isotropic property (mainly due to elastin) dominates at lower blood pressures while the anisotropic property (mainly due to collagen) involvement becomes gradually larger with increasing pressure (Marsh et al., 2004). It has been suggested that anisotropy may affect aneurysm formation, indicating that models of the vascular wall that only take isotropy into account are insufficient for simulations (Rodriguez et al., 2008).

Measuring isotropic and anisotropic properties is difficult *in vivo*. We developed an explorative computer model which allows the computation of wall stress as well as isotropic and anisotropic components in the circumferential ( $\sigma_{\theta }^{iso}$ ; $\sigma_{\theta }^{aniso}$ ) and longitudinal directions ( $\sigma_{z}^{iso}$ ; $\sigma_{z}^{aniso}$ ) (Stalhand, 2009; Gade et al., 2019). By modeling the vessel wall, mechanical properties and constituents can be explored in both extreme and average states. The model may also serve as a potential tool for aneurysm assessment. The aim of this study was to investigate PWS of the AA as well as isotropic and anisotropic stress components in healthy human aortas with respect to age and sex.

## Material and methods

### Study subjects

Thirty healthy, non-smoking adult (males = 15) volunteers were included in the study and divided in two age-groups: young (23–30 years, n = 10) and elderly (41–72 years, n = 20). Inclusion was based on the absence of a history of cardiopulmonary disease, diabetes, or regular medication. No woman was subjected to oestrogen replacement therapy. The data-set has previously been analysed from a different perspective (Astrand et al., 2011) The monitoring of diameter and pressure changes is briefly outlined below and has been described elsewhere (Astrand et al., 2011; Karlsson et al., 2022).

### Non-invasive monitoring of diameter changes

For non-invasive monitoring of pulsatile diameter changes in the distal AA, an ultrasound echo-tracking system (Diamove, Teltec AB, Lund, Sweden) was used (Astrand et al., 2011). It was interfaced with a real-time ultrasound scanner (EUB-240; Hitachi, Tokyo, Japan) and fitted with a 3.5 MHz linear array transducer. The instrument was equipped with dual echo-tracking loops, enabling the simultaneous tracking of separate echoes from the proximal and distal vessel walls. The repetition frequency (sampling frequency) of the echo-tracking loop was 870 Hz, temporal resolution was 1.2 ms and the smallest detectable movement was 7.8 µm. For static (end diastolic and systolic) aortic diameter and for pulsatile diameter change, the coefficient of variation was 5% and 16%, respectively.

### Invasive blood pressure measurements

Blood pressure in the AA was measured with a 3F (SPC 330A) or 4F (SPC 340) micromanometer tip catheter (Millar Instruments, Houston, Texas) in half of the volunteers and a fluid-filled catheter system (pressure monitoring kit DTX+ with a R.O.S.E.; Viggo Spectramed, Oxnard California) in the other half due to availability. The frequency response of the Millar catheter (flat range to 10 kHz) was higher than in the fluid-filled system [flat range 35 Hz (3 dB)]. However, the amplitude was identical when the graph of one cardiac cycle from each pressure system created by a Blood System Calibrator (Bio Tech Model 601 A; Bio Tech Burlington, Vt.) was superimposed on each other. No difference in maximal SBP or minimal diastolic blood pressure (DBP) was observed.

**FIGURE 1:**
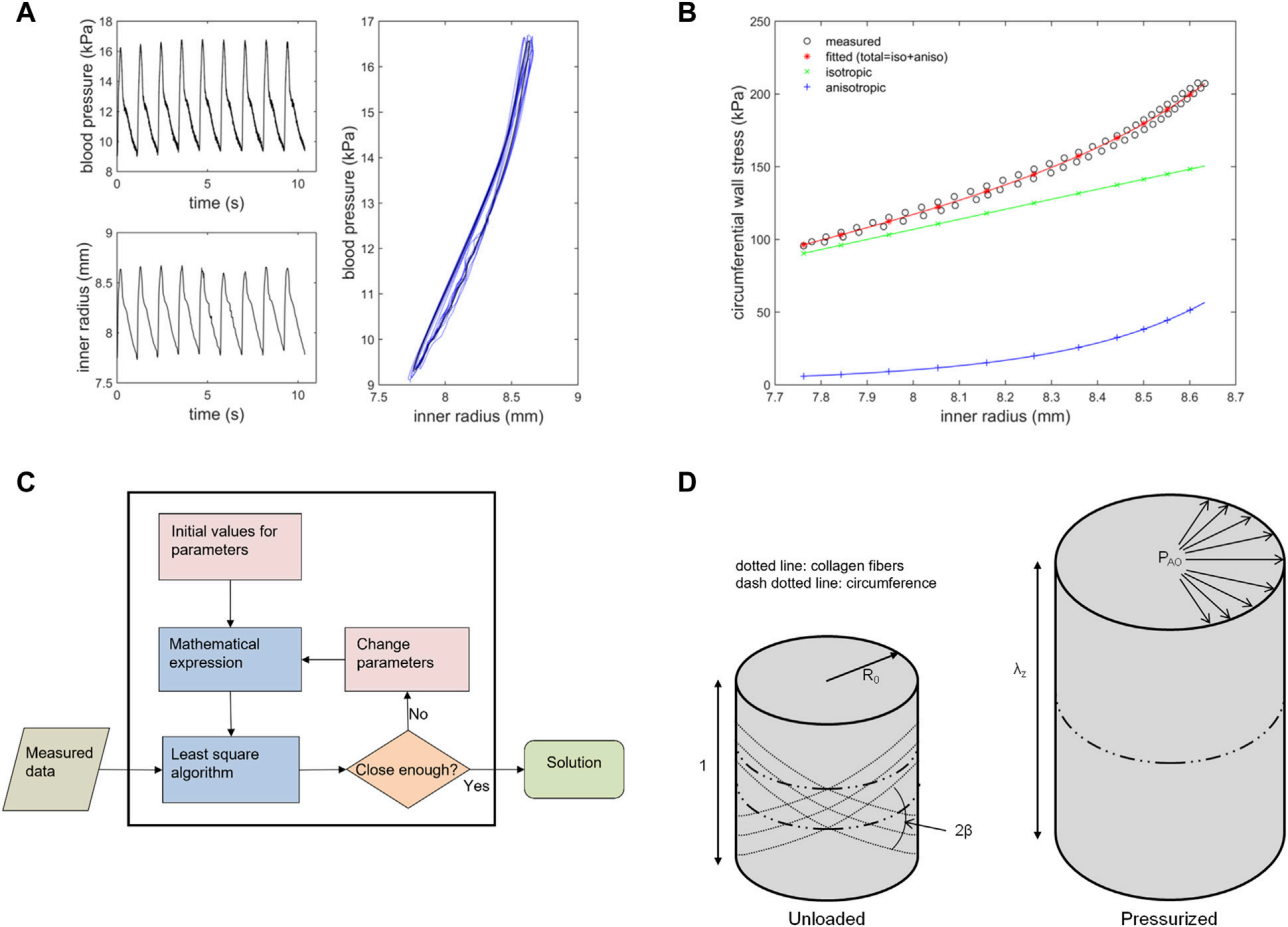
A and B illustrate measured data from the abdominal aorta and identification results from PIMMP. C and D show the identification algorithm and definitions of some model parameters **(A)** Measured blood pressure (upper left panel) and inner radius (lower left panel) for a young male. Right panel shows the pressure-radius response (blue) and the used post processed average signal (black) **(B)** Wall stresses vs. inner radius in the circumferential direction for a young male. Measured data (black) with resulting fitted curve from PIMMP representing total stress (red) which is the sum of the isotropic (green) and anisotropic (blue) components **(C)** The identification algorithm used to compute the material parameters in the abdominal aorta, see text for description **(D)** An overview of how model parameters *β*, *R*_*0*_ and *λ*_*z*_ are defined. The left cylinder represents the stress and strain free, unloaded state with unit length; the right cylinder represents the pressurized state. P_AO_ = Pressure in the abdominal aorta, PIMMP = parameter identification method for mechanical parameters.

The simultaneous monitoring of arterial blood pressure and vessel diameter was possible through a data acquisition system containing a personal computer type 386 (Express, Tokyo, Japan) and a 12-bit analogue-to-digital converter (Analog Devices, Norwood, Massachusetts). Example of acquired data can be found in Figure 1A.

### Stress

**FIGURE 2:**
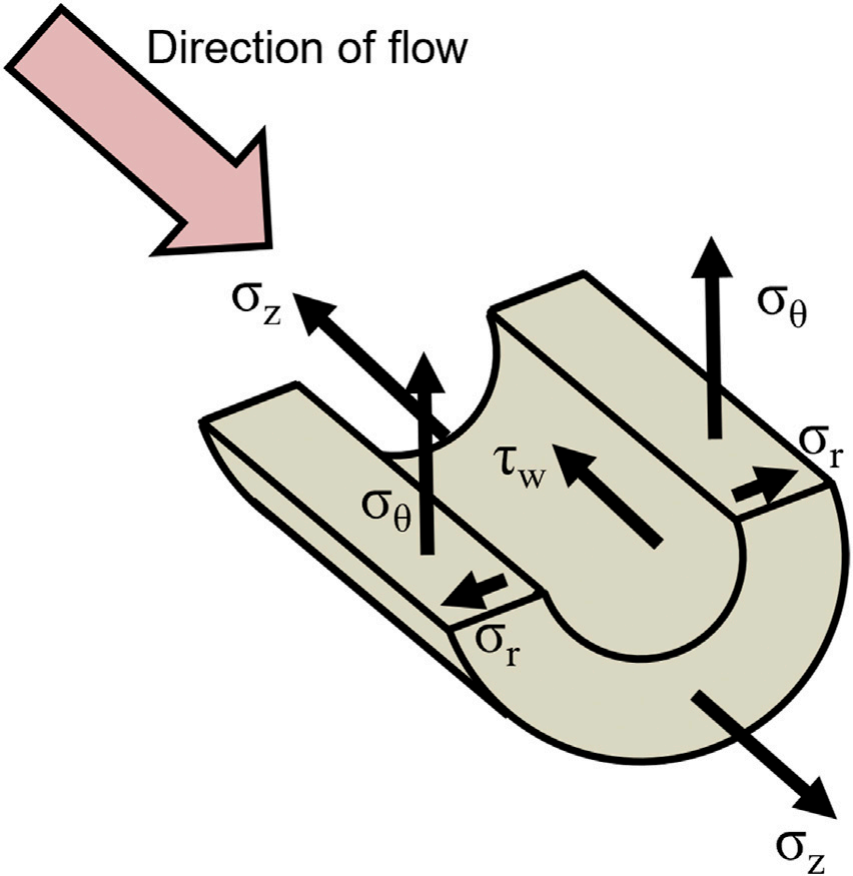
Blood pressure and flow in an artery yields the three primary loads acting on the vessel wall: pressure-induced circumferential wall stress $\sigma_{\theta }$ , axial load-induced longitudinal wall stress $\sigma_{z}$ and flow-induced wall shear stress $\tau_{w}$ . Radial wall stress $\sigma_{r}$ is small compared to the circumferential and longitudinal stresses and is neglected in this study. Note, arrows are only schematic, no correlation with magnitude intended.

Stress is defined as force divided by the area on which the force acts. It is measured in Pascal (Pa). In general, force has two components that are oriented either perpendicular (normal) or parallel to the surface. The force from the blood pressure acting on the intraluminal face of the vessel is an example of perpendicular force, giving rise to circumferential (hoop), longitudinal (axial), and radial stress in the vessel wall. Blood flow acting on endothelial cells is a parallel force that generates wall shear stress. Circumferential, longitudinal, and shear stresses are considered the most important hemodynamic forces influencing vessel size and morphology (Humphrey, 2008). Radial stress is small compared to circumferential and longitudinal stresses (Fung, 1997) and was, therefore, neglected in this study. Because wall shear stress is very small (1–5 Pa) and smooth muscles are considered being in a non-contractile state, it is insignificant, outside the scope of this study, and will not be further considered (Figure 2).

### The mechanical model and the identification of model parameters

#### Equilibrium stress

The pressurized (physiological) abdominal aorta is regarded as a thin-walled incompressible cylinder of length *l* and inner radius *r*_*0*_ and a wall thickness *h*. The lumen pressure *P* is applied to the inner boundary, whereas the outer boundary is stress free (Figure 1D). The membrane stresses in circumferential and longitudinal directions are given by Laplace’s law and can be written, respectively [8]:

$$\sigma_{\theta }^{lp}=\frac{4\pi r_{0}^{2}+A}{2A}P,\sigma_{z}^{lp}=\frac{\pi r_{0}^{2}P+F}{A}$$ (1)

where *A* is the arterial cross-sectional area, and *F* is the *in situ* axial force. In the equations above, wall thickness has been replaced for the cross-sectional area using $A=2\pi r_{0}h$ . Since the cross-sectional area is not available for the dataset in use, it is calculated from Åstrand et al. (2005). The cross-sectional area in the abdominal aorta is given by A=19.60+0.80*age (males) and A=20.52+0.56*age (females), where *age* is in years and *A* in mm^2^ (Astrand et al., 2005; Stalhand, 2009).

Assuming the axial force and axial stretch are constant and independent of the internal pressure, and the ratio between the longitudinal and circumferential stress is known at one internal pressure $\tilde{P}$ with a corresponding $\tilde{r}$ , the membrane stress can be computed from Laplace’s law in both circumferential and longitudinal directions using measured pressure and diameter (Stalhand, 2009). The axial force can be determined explicitly by applying the second assumption above with the stress ratio taken to be $\gamma =\sigma_{z}^{lp}/\sigma_{\theta }^{lp}=0.59$ at $\tilde{P}=13.3$ kPa, following Schulze-Bauer and Holzapfel (Schulze-Bauer and Holzapfel, 2003):

$$F=\pi {\tilde{r}}_{0}^{2}\tilde{P}\left(2\gamma +\frac{\gamma A}{2\pi {\tilde{r}}_{0}^{2}}-1\right)$$ (2)

Note that membrane stresses according to Laplace’s law do not depend on the material properties of the vessel. They are only functions of the applied load (pressure) and geometry (diameter), and, as a consequence, the membrane stress becomes statically determined (Stalhand, 2009).

#### Constitutive stress

The mechanical model used herein is based on a standard Holzapfel-Gasser-Ogden (HGO) non-linear material model with a neo-Hookean matrix reinforced by a two-family fiber structure; the strain energy function suggested by Holzapfel et al. (Holzapfel et al., 2000):

$$\psi =\psi_{iso}+\psi_{aniso}=c\left(I_{l}-3\right)+\frac{k_{I}}{k_{2}}\left({\mathrm{e}}^{k_{2}{\left(I-1\right)}^{2}}-1\right)$$ (3)

where *c*, *k*_*1*_, *k*_*2*_ > 0 and the invariants are $I_{1}=\lambda_{\theta }^{2}+\lambda_{z}^{2}+{\left(\lambda_{\theta }^{2}\lambda_{z}^{2}\right)}^{-1}$ and $I=\lambda_{\theta }^{2}\cos^{2}\beta +\lambda_{z}^{2}\sin^{2}\beta$ . The circumferential and axial stretches are denoted by $\lambda_{\theta }$ and $\lambda_{z}$ , respectively. The former stretch is computed in the mid-wall and given by $\lambda_{\theta }=\frac{R_{0}}{r_{0}}\frac{4\pi r_{0}^{2}+A}{4\pi R_{0}^{2}+\lambda_{z}A}$ , where $R_{0}$ is the inner radius of the unloaded and stress-free state. The latter stretch $\lambda_{z}$ describes the arterial length *in situ* relative to the unloaded state. Note that both $R_{0}$ and $\lambda_{z}$ define the geometry of the unloaded artery and cannot be obtained directly in *in vivo* experiments. Instead, they are included in the identification and obtained simultaneously with the parameter describing the material. The circumferential and longitudinal stresses can be computed as:

$$\sigma_{\theta }=-p+\lambda_{\theta }\frac{\partial \psi }{\partial \lambda_{\theta }}=\sigma_{\theta }^{iso}+\sigma_{\theta }^{aniso}=2C\left[\lambda_{\theta }^{2}-\frac{1}{{\left(\lambda_{\theta }\lambda_{z}\right)}^{2}}\right]+4k_{1}\left(I-1\right){\mathrm{e}}^{k_{2}{\left(I-1\right)}^{2}}\lambda_{\theta }^{2}\cos^{2}\beta$$ (4)

$$\sigma_{z}=-p+\lambda_{z}\frac{\partial \psi }{\partial \lambda_{z}}=\sigma_{z}^{iso}+\sigma_{z}^{aniso}=2C\left[\lambda_{z}^{2}-\frac{1}{{\left(\lambda_{\theta }\lambda_{z}\right)}^{2}}\right]+4k_{1}\left(I-1\right){\mathrm{e}}^{k_{2}{\left(I-1\right)}^{2}}\lambda_{z}^{2}\sin^{2}\beta$$ (5)

The arbitrary multiplier −p in Eqs. 4, 5 arises due to incompressibility and is computed using that the transmural (radial) stress $\sigma_{r}$ is negligible for thin-walled tubes. For the chosen strain energy, the transmural stress becomes $\sigma_{r}=-p+2c{\left(\lambda_{\theta }\lambda_{z}\right)}^{-2}\approx 0$ (Stalhand, 2009).

#### Parameter identification

The model includes six unknown parameters $R_{0},\lambda_{z},c,k_{1},k_{2},\beta$ which must be identified. This is done using a non-linear least-squares fitting of the equilibrium stresses in Eq. 1 and the constitutive stresses in Eqs. 4, 5. The error function is given by:

$$\phi \left(\kappa \right)=\overset{N}{\underset{n=1}{\sum }}\left\{{\left[\sigma_{\theta }\left(\kappa ,r_{0,n}\right)-\sigma_{\theta }^{lp}\left(\kappa ,r_{0,n},P_{n}\right)\right]}^{2}+{\left[\sigma_{z}\left(\kappa ,r_{0,n}\right)-\sigma_{z}^{l_{P}}\left(\kappa ,r_{0,n},P_{n}\right)\right]}^{2}\right\}$$ (6)

where $\kappa =\left(R_{0},\lambda_{z},c,k_{1},k_{2},\beta \right)$ is the parameter vector, $r_{0,n}$ and $P_{n}$ are the inner radius and pressure at sample n , and *N* is the total number of samples. The model parameters are computed as the solution to the minimization problem:

$$\left\{\begin{array}{l} \min_{\kappa }\phi \left(\kappa \right)\\ subject\;to:\underline{\kappa_{j}}\leq \kappa_{j}\leq \bar{\kappa_{j}} \end{array}\right.$$

where $\kappa_{j}$ is the *j* ^th^ component in the parameter vector κ , and $\underline{\kappa_{j}}$ and $\bar{\kappa_{j}}$ denote its lower and upper bounds. The minimization fitting ranges are based on experimental observations and chosen in order to avoid activation during minimization (Table 1). The lower boundary for $\lambda_{z}$ is set to one to avoid buckling.

**TABLE 1:** Fitting ranges of the model parameters.

	κ_min_	κ_max_
*c* (kPa)	1*10^–4^	5*10^5^
*k*_*1*_ (kPa)	1*10^–4^	5*10^5^
*k*_*2*_ (−)	1*10^–4^	1*10^3^
β (°)	1*10^–6^	90
*R*_*0*_ (mm)	1	20
*λ*_*z*_ (−)	1	5

#### Identification routine

The mechanical model with parameter identification routine consists of two steps: first, a signal processing routine and, second, a parameter identification routine including a non-linear mechanical model (Stalhand, 2009; Astrand et al., 2011). In the first step, the measured pressure and radius signals consisted of approximately 8–10 cycles (heartbeats) and were imported to MATLAB. The processed data were lowpass filtered with a fourth-order Butterworth filter with a cut-off frequency of 15 Hz for noise reduction. Furthermore, the data were automatically adjusted for time delays from the measurement setup. Finally, pressure and radius were averaged over cycles (Stalhand, 2009). In the second step (parameter identification), the model parameters were identified through a non-linear curve fitting of the model response to the measured pressure-radius loop using an iterative scheme. The identification algorithm includes the following steps (Figure 1C).

1) Stresses in the arterial wall were computed by Laplace’s law in both circumferential and longitudinal directions using the pressure-radius loop (Stalhand, 2009) together with an estimation of the aortic wall cross-sectional area (*A*) of the aorta as described above (Astrand et al., 2005; Astrand et al., 2011).
2) A second set of wall stresses was computed using a model based on a standard Holzapfel-Gasser-Ogden non-linear continuum mechanical model (Eq. 3) (Holzapfel et al., 2000). These model stresses are dependent on six model parameters which describe the material characteristics and the *in situ* pre-stress of the aortic wall and are described below. (Fung, 1997; Stalhand et al., 2004; Horny et al., 2012).
3) By comparing the Laplace stresses in step 1) to the model stresses from step 2), an estimate of the error (difference) was obtained by summing the squared residuals (Eq. 6) (Stalhand, 2009). If the error between two consecutive iterations exceeds a pre-set tolerance (typically 10^–5^), the parameters values were updated using standard identification techniques and steps 2) and 3) were repeated.

Six model parameters were identified and they describe the material characteristics (*c*, *k*_*1*_, *k*_*2*_, *β*) and geometrical properties (*R*_*0*_ and *λ*_*z*_) of the stress free aortic wall (Astrand et al., 2011). Values are presented in Table 4. The parameters are.

• *c* (Pa)—relates to the stiffness of the isotropic constituents in the vascular wall, mainly elastin.
• *k*_*1*_ (Pa)—relates to the stiffness of the anisotropic constituents in the vascular wall, mainly collagen.
• *k*_*2*_ (dimensionless)—reflects the crimpling or folding, cross-linking and entanglement of collagen.
• *β* (°)—the angle between the circumferential direction and the principal (mean) fiber direction in the unloaded configuration (Figure 1D).
• *R*_*0*_ (mm)—the radius in a strain and stress free (*ex situ*) unloaded configuration (Figure 1D).
• *λ*_*z*_ (dimensionless)—axial stretch between the *in situ* configuration and the (*ex situ*) stress free configuration (Figure 1D).

Computed circumferential stress with the result from the identification routine is illustrated in (Figure 1B).

### Computed variables

Computed stress can be separated into isotropic and anisotropic components in both the circumferential ( $\sigma_{\theta }=\sigma_{\theta }^{iso}+\sigma_{\theta }^{aniso}$ ) and longitudinal directions ( $\sigma_{z}=\sigma_{z}^{iso}+\sigma_{z}^{aniso}$ ) (Stalhand, 2009; Astrand et al., 2011). Isotropy and anisotropy are directional properties linked to the constituent’s orientation but independent of the material shape and volume. For the vascular wall, the isotropic and anisotropic components primarily reflect structures such as elastin and collagen, respectively (Holzapfel et al., 2000; Humphrey, 2002).

Six different stresses were computed and investigated with PIMMP (Figure 1B): i) total circumferential wall stress; ii) isotropic circumferential wall stress component; iii) anisotropic circumferential wall stress component; iv) total longitudinal wall stress; v) isotropic longitudinal wall stress component; and vi) anisotropic longitudinal wall stress component. The isotropic and anisotropic wall stress components reflect the behavior of isotropic and anisotropic constituents of the arterial wall, respectively. Total stress is used to denote the result from adding isotropic and anisotropic stress ( $\sigma^{tot}=\sigma^{iso}+\sigma^{aniso}$ ).

Pulse pressure was calculated as SBP—DBP. Pulsatile stress was calculated as ∆σ=σ@SBP−σ@DBP in circumferential and longitudinal directions, and their respective isotropic and anisotropic components. All pressures and stresses are in Pascals (Pa).

Fractions of total stress for isotropic and anisotropic components at SBP and DBP were defined as ${F\sigma }_{n}^{m}=\sigma_{n}^{m}/\sigma_{n}^{tot}$ while fractions of pulsatile stress were defined as ${F∆\sigma }_{n}^{m}={∆\sigma }_{n}^{m}/{∆\sigma }_{n}^{tot}$ , *m* = [isotropic or anisotropic] and *n* = [θ or z]. This constitutes load bearing fraction of material with isotropic and anisotropic properties.

In males and females, all variables were computed at SBP and DBP in both the young and elderly groups. Total circumferential and longitudinal stresses were also computed at MAP. MAP was calculated as DBP + 1/3 * (SBP-DBP), unit Pa. To convert Pa to mmHg, 1 mmHg = 133.32 Pa, was used. Peak wall stress is sometimes used to denote total circumferential or longitudinal stress at SBP.

The coefficient of determination (*R* ^*2*^) was computed as a tool to evaluate the overall fit between stress curves computed according to Laplace’s law and PIMMP:

$$R^{2}=1-\frac{\sum_{n=1}^{N}{\left(y_{n}-{\hat{y}}_{n}\right)}^{2}}{\sum_{n=1}^{N}{\left(y_{n}-\bar{y}\right)}^{2}}$$ (7)

where $y_{n}$ is results from Laplace’s law, ${\hat{y}}_{n}$ is results from PIMMP, $\bar{y}$ is the mean of results from Laplace’s law and *N* is the total number of samples.

A minor sensitivity test was conducted using *R* ^*2*^. A radius and a pressure were computed, representing all participants, and used to produce a baseline of material parameters and *R* ^*2*^. Model parameters were then increased with one percentage point (1%), one at a time, and new perturbed stresses were computed and compared to baseline through a new *R* ^*2*^. In total (not counting baseline), 6 *R* ^*2*^ were computed, representing each altered model parameter.

### Statistics

Maximum and minimum values of a variable were calculated at SBP and DBP, respectively. Arithmetic mean and standard deviation (SD) were calculated for all variables and are expressed as mean ± SD, if not otherwise stated. Sex and age groups were compared using a two-way ANOVA with complementing general linear models, as suggested by Field (Field, 2013). Bonferroni correction was used when multiple comparisons were made. Linear regression analysis with the Pearson correlation coefficient (*r* ^*2*^) was used to assess dependence of all parameters on age within each sex. *p* < 0.05 was considered significant in the ANOVA and general linear model as well as in the linear regression analysis. Significance testing is used for a descriptive purpose.

### Software

MATLAB (MathWorks, Natick, MA, U.S), version 8.4 (R2014b) was used for computations. IBM SPSS Statistics, version 27 (IBM Corporation, Armonk, NY, US), was used for statistical analysis.

## Results

During the statistical analysis, it was clear that the small population offered a wide variation in measured and computed values, precluding the achievement of statistically significant results using three age groups as in Åstrand et al. (2011). Therefore, we decided to regroup the population into two age groups, above and below 40 years of age. In the reporting of results we decided that statistically non-significant results would be omitted.

### Baseline patient characteristics and model parameters

Table 2 shows the baseline clinical data and Table 3 shows AA diameters and blood pressures for the study population. Males had larger abdominal aortic diameters than females (*p* < 0.05). Changes in the pulsatile diameter of the AA (Δ*D*) and blood pressure did not differ by sex. Young males had lower systolic blood pressure (SBP) and mean arterial pressure (MAP) values, smaller AA diameters but larger Δ*D* than elderly males (*p* < 0.05). Young females had smaller AA diameters but larger Δ*D* than elderly females (*p* < 0.05).

**TABLE 2:** Characteristics of the studied population.

	Young 23–30 years		Elderly 41–72 years	
	Male, n = 5	Female, n = 5	Male, n = 10	Female, n = 10
Age, yr	24.8 ± 1.9	25.4 ± 2.8	58.6 ± 12.1	59.0 ± 10.6
Height, cm	177 ± 8.9	171 ± 8.9	181 ± 6.3	170 ± 6.3^¤^
Weight, kg	71.4 ± 8.7	59.0 ± 9.4	86.0 ± 12.0^†^	65.8 ± 8.5^#^
BMI, kg/m^2^	22.6 ± 0.9	20.1 ± 2.0	26.2 ± 2.5^†^	22.8 ± 3.5^*^
BSA, m^2^	1.88 ± 0.18	1.69 ± 0.18^*^	2.08 ± 0.16^†^	1.76 ± 0.12^#^

Data are presented as mean ± SD. BMI: body mass index, BSA: body surface area.

^*^ *p* < 0.05.

^¤^ *p* < 0.01.

^#^ *p* < 0.001, between males and females within an age group.

^†^ *p* < 0.05, between elderly and young within males.

**TABLE 3:** Abdominal aortic pressure and diameter.

	Young 23–30 years		Elderly 41–72 years	
	Male, n = 5	Female, n = 5	Male, n = 10	Female, n = 10
SBP, mmHg	114 ± 12	119 ± 14	134 ± 19^†^	125 ± 12
DBP, mmHg	62 ± 8	66 ± 9	71 ± 9	65 ± 6
MAP, mmHg	79 ± 9	84 ± 11	92 ± 11^†^	85 ± 7
PP, Δ*P*, mmHg	52 ± 5	52 ± 5	63 ± 15	60 ± 9
Diameter SBP, mm	15.9 ± 1.2	15.1 ± 0.8	20.0 ± 1.7^†^	16.8 ± 1.8^*^
Diameter DBP, mm	13.8 ± 1.4	13.3 ± 1.4	19.0 ± 1.9^†^	15.8 ± 1.9^†^ ^,^ ^*^
Δ*D*, mm	2.14 ± 0.32	1.79 ± 0.75	0.96 ± 0.41^†^	0.98 ± 0.44^†^

Data are presented as mean ± SD. SBP: systolic blood pressure, DBP: diastolic blood pressure, MAP: mean arterial pressure, PP, Δ*P*: pulse pressure, Diameter SBP: diameter at systolic blood pressure, Diameter DBP: diameter at diastolic blood pressure, Δ*D*: Diameter SBP−Diameter DBP.

^*^ *p* < 0.05, between males and females in the elderly age group.

^†^ *p* < 0.05, between elderly and young within respective sex.

Table 4 shows model parameters. There were no differences when comparing model parameters between males and females. Model parameters are in agreement with results published by Åstrand et al. (2011) (Astrand et al., 2011). The value of the standard deviation is an order of magnitude higher than the corresponding mean value for stiffness parameters compared with geometrical parameters. We propose this could be an expression of variation within the population identified by the model.

**TABLE 4:** Model parameters.

	Male	Female
*c* (kPa)	131.50 ± 90.00	101.65 ± 63.75
*k*_*1*_ (kPa)	14.18 ± 20.94	8.64 ± 10.00
*k*_*2*_ (−)	196.76 ± 264.22	134.95 ± 175.15
β (°)	42.38 ± 4.38	42.31 ± 3.82
*R*_*0*_ (mm)	7.80 ± 1.78	6.77 ± 1.23
*λ*_*z*_ (−)	1.03 ± 0.02	1.03 ± 0.02

Data are presented as mean ± SD.

The computed coefficient of determination (*R* ^*2*^) showed an overall good agreement between stress according to Laplace’s law and constitutive stress for all subjects: both circumferential and longitudinal stress had an *R* ^*2*^ of 0.98 ± 0.01.

### Aortic wall stress computed with PIMMP

**FIGURE 3:**
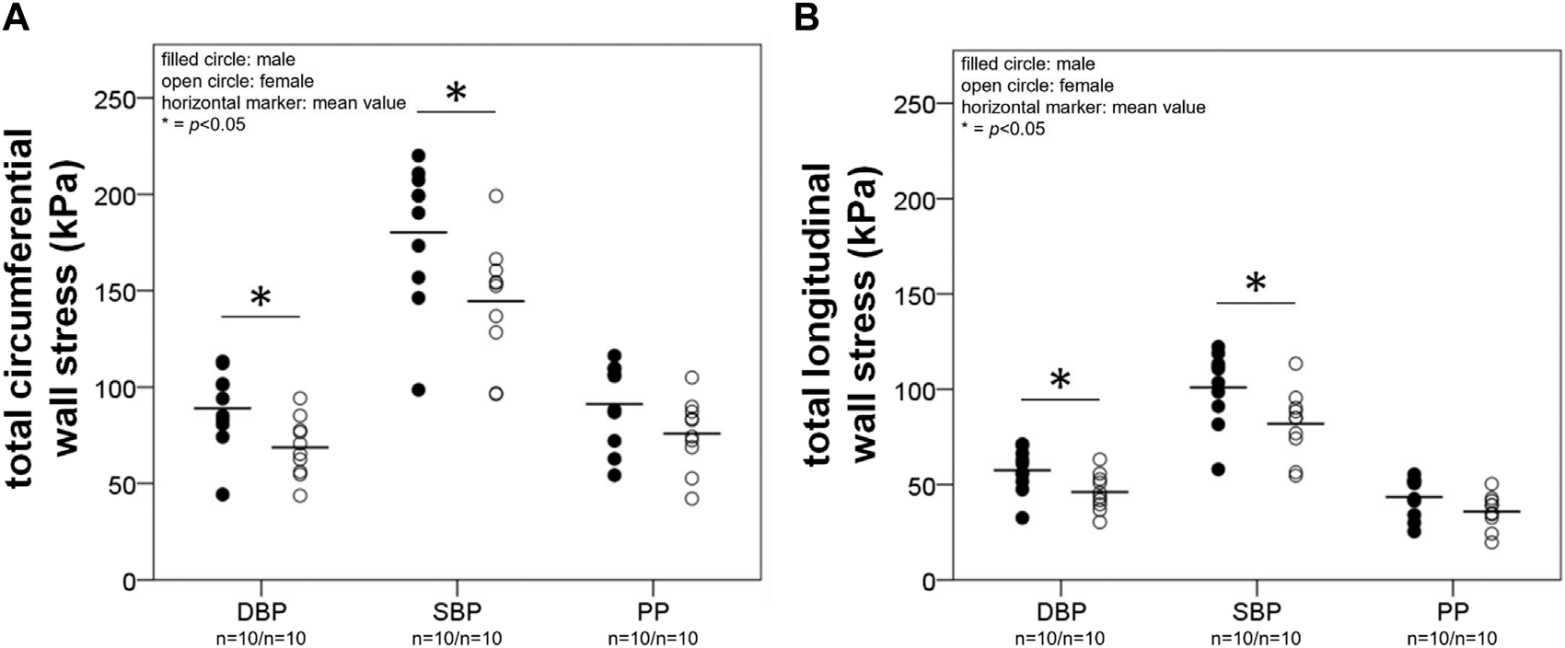
A and B depict results from the calculation of total wall stress. Total circumferential **(A)** and longitudinal **(B)** stress ( $\sigma_{\theta }^{tot}$ ; $\sigma_{z}^{tot}$ ) are given at diastolic blood pressure (DBP), systolic blood pressure (SBP) and pulse pressure (PP) for the elderly group. Males had higher $\sigma_{\theta }^{tot}$ as well as $\sigma_{z}^{tot}$ than females at DBP and SBP. Stress at PP denotes pulsatile stress ( ∆σ ), i.e., stress at SBP minus stress at DBP.

**Total stress**. Figure 3 shows total circumferential (A) and longitudinal (B) wall stress at DBP, SBP, and pulse pressure (PP) interval in males and females for the elderly group. Stress at PP denotes pulsatile stress ( ∆σ ), i.e., stress at SBP minus stress at DBP. Males had higher total circumferential and longitudinal wall stress ( $\sigma_{\theta }^{tot}$ ; $\sigma_{z}^{tot}$ ) than females at DBP (89.0 ± 20.6 vs. 68.7 ± 15.3 kPa, *p* < 0.05 and 57.5 ± 11.7 vs. 46.0 ± 9.7 kPa, *p* < 0.05, respectively), as well as at SBP (180.2 ± 37.3 vs. 144.50 ± 31.4 kPa, *p* < 0.05 and 101.0 ± 19.7 vs. 82.0 ± 17.6 kPa, *p* < 0.05, respectively) and at MAP (112.8 ± 25.1 vs. 88.9 ± 18.9 kPa, *p* < 0.05 and 68.4 ± 13.8 vs. 55.1 ± 11.4 kPa, *p* < 0.05). $\sigma_{\theta }^{tot}$ appeared to be higher in elderly males compared to young males at DBP (89.0 ± 20.6 vs. 69.3 ± 21.0 kPa, *p* = 0.07). $\sigma_{z}^{tot}$ appeared to be lower in elderly females compared to young females at DBP (46.0 ± 9.7 vs. 52.7 ± 9.1 kPa, *p* = 0.08).

In females pulsatile total circumferential and longitudinal stress correlated negatively with age (*R* ^*2*^ = 0.27, *p* < 0.05 and *R* ^*2*^ = 0.29, *p* < 0.05, respectively), while in males there were no correlations with age.

**Aortic isotropic and anisotropic stress in the circumferential direction**. In the elderly group, males had a higher isotropic circumferential wall stress component ( $\sigma_{\theta }^{iso}$ ) than females at DBP (83.0 ± 20.1 vs. 65.0 ± 17.2 kPa, *p* < 0.05). $\sigma_{\theta }^{iso}$ in elderly males was higher than in young males at DBP (83.0 ± 20.1 vs. 56.1 ± 21.2 kPa, *p* < 0.05). $\sigma_{\theta }^{iso}$ in females, showed no difference between the young and the elderly group.

**FIGURE 4:**
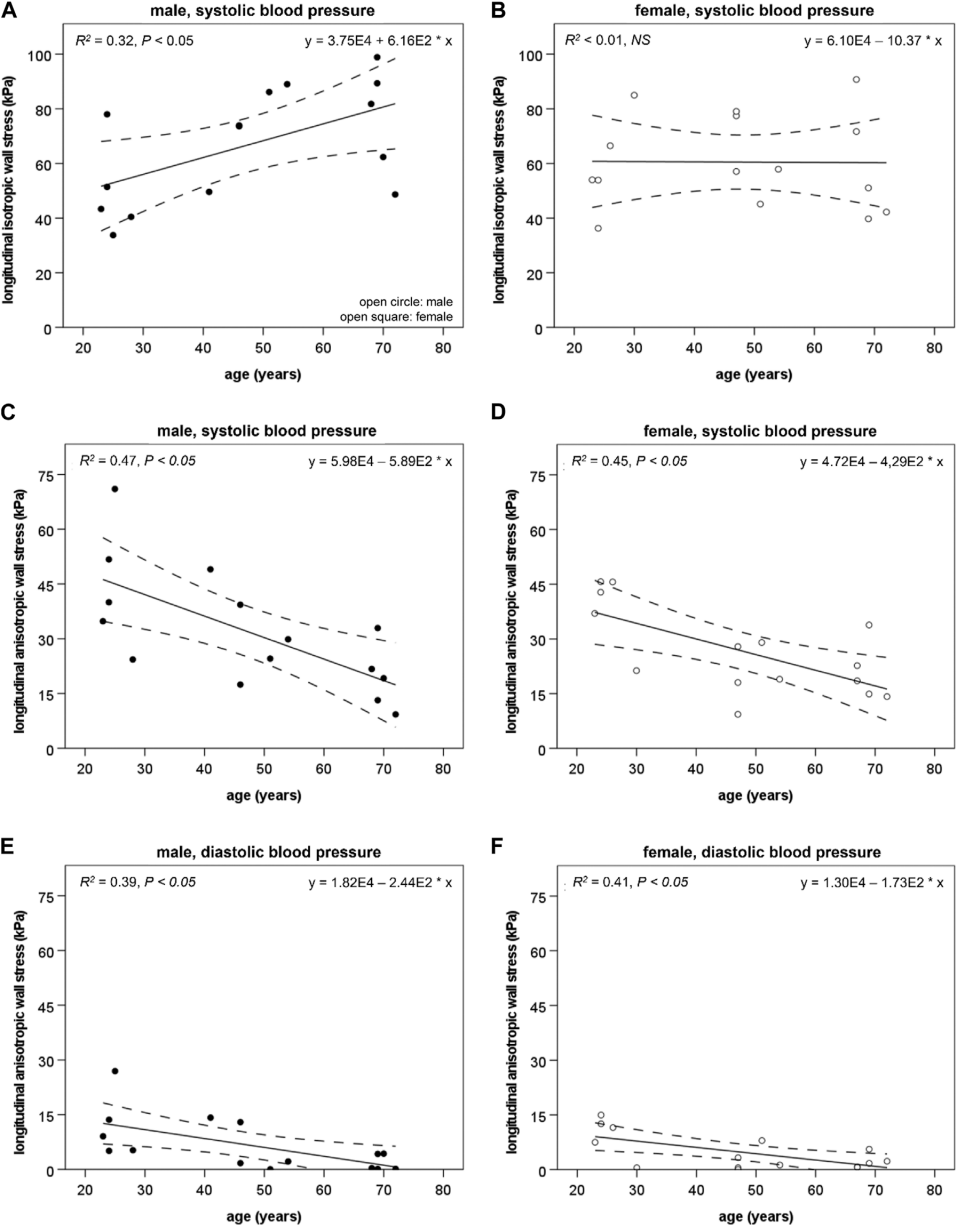
The longitudinal isotropic wall stress component ( $\sigma_{z}^{iso}$ ) is depicted at systolic blood pressure (SBP) in the male **(A)** and female **(B)** abdominal aorta. In males, $\sigma_{z}^{iso}$ increases with age (*R* ^*2*^ = 0.32, *p* < 0.05), while in females, $\sigma_{z}^{iso}$ and age did not correlate (*R* ^*2*^ < 0.01, NS). The longitudinal anisotropic wall stress component ( $\sigma_{z}^{aniso}$ ) is shown at SBP and diastolic blood pressure (DBP) for the abdominal aorta in males **(C, E)** and females **(D, F)**. $\sigma_{z}^{aniso}$ decreases in males and females at SBP (*R* ^*2*^ = 0.47, *p* < 0.01 and *R* ^*2*^ = 0.45, *p* < 0.01, respectively) and DBP (*R* ^*2*^ = 0.39, *p* < 0.05 and *R* ^*2*^ = 0.41, *p* < 0.01, respectively). Dashed lines mark a 95% confidence interval.

**Aortic isotropic and anisotropic stress in the longitudinal direction**. The isotropic longitudinal wall stress component ( $\sigma_{z}^{iso}$ ) was higher in elderly males than in young males at SBP (75.4 ± 17.1 vs. 49.4 ± 17.2 kPa, *p* < 0.05) and DBP (53.5 ± 12.1 vs. 36.3 ± 13.6 kPa, *p* < 0.05); this trend was also true for the pulsatile isotropic longitudinal wall stress component (21.9 ± 7.6 vs. 13.1 ± 4.0 kPa, *p* < 0.05). Figures 4A, B show $\sigma_{z}^{iso}$ in males and females at SBP. In males; $\sigma_{z}^{iso}$ and age, as well as pulsatile isotropic longitudinal wall stress component and age, correlated positively (*R* ^*2*^ = 0.32, *p* < 0.05 and *R* ^*2*^ = 0.27, *p* < 0.05, respectively). In females, $\sigma_{z}^{iso}$ and age did not correlate (*R* ^*2*^ < 0.01, NS).

The anisotropic longitudinal wall stress component ( $\sigma_{z}^{aniso}$ ) at SBP and DBP as well as pulsatile anisotropic longitudinal wall stress component ( ${∆\sigma }_{z}^{aniso}$ ) were lower in the elderly compared with young males ( $\sigma_{z}^{aniso}$ : SBP 25.6 ± 12.3 vs. 44.4 ± 17.9 kPa, *p* < 0.005 and DBP 4.0 ± 5.3 vs. 12.0 ± 9.0 kPa, *p* < 0.01; ${∆\sigma }_{z}^{aniso}$ : 21.6 ± 8.2 vs. 32.4 ± 10.0 kPa, *p* < 0.01). Figures 4C, E show $\sigma_{z}^{aniso}$ in males at SBP and DBP. $\sigma_{z}^{aniso}$ correlated negatively with age in males at SBP and DBP (*R* ^*2*^ = 0.47, *p* < 0.01 and *R* ^*2*^ = 0.39, *p* < 0.05, respectively). ${∆\sigma }_{z}^{aniso}$ , also correlated negatively with age in males (*R* ^*2*^ = 0.44, *p* < 0.01).

$\sigma_{z}^{aniso}$ was lower in elderly compared with young females at SBP (20.7 ± 7.6 vs. .38.5 ± 10.2 kPa, *p* < 0.05) and DBP (2.4 ± 2.6 vs. 9.4 ± 5.7 kPa, *p* < 0.05); this trend was also true for ${∆\sigma }_{z}^{aniso}$ (18.3 ± 5.9 vs. 29.1 ± 5.0 kPa, *p* < 0.05). Figures 4D, F shows $\sigma_{z}^{aniso}$ in females at SBP and DBP. $\sigma_{z}^{aniso}$ correlated negatively with age in females at SBP and DBP (*R* ^*2*^ = 0.45, *p* < 0.01 and *R* ^*2*^ = 0.41, *p* < 0.01, respectively). ${∆\sigma }_{z}^{aniso}$ also correlated negatively with age in females (*R* ^*2*^ = 0.40, *p* < 0.05).

**FIGURE 5:**
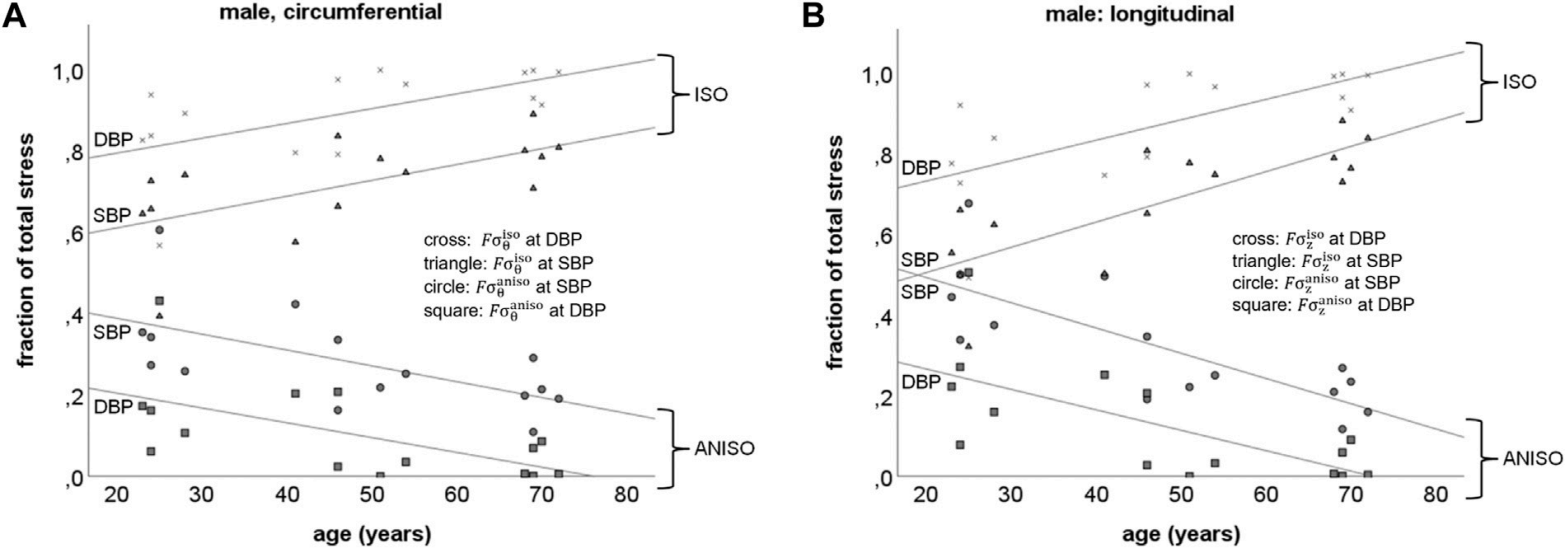
Schematic representation of the load bearing fractions of material with isotropic and anisotropic properties in the abdominal aorta at systolic (SBP) and diastolic (DBP) blood pressures in males, in the circumferential (A: ${F\sigma }_{\theta }^{iso}$ ; ${F\sigma }_{\theta }^{aniso}$ ) and in the longitudinal (B: ${F\sigma }_{z}^{iso}$ ; ${F\sigma }_{z}^{aniso}$ ) direction **(A) DBP**: ${F\sigma }_{\theta }^{iso}$ *R* ^*2*^ = 0.36, *p* < 0.05; ${F\sigma }_{\theta }^{aniso}$ *R* ^*2*^ = 0.36, *p* < 0.05; **SBP**; ${F\sigma }_{\theta }^{iso}$ *R* ^*2*^ = 0.38, *p* < 0.05; ${F\sigma }_{\theta }^{aniso}$ *R* ^*2*^ = 0.38, *p* < 0.05 **(B) DBP**: ${F\sigma }_{z}^{iso}$ *R* ^*2*^ = 0.46, *p* < 0.01 and ${F\sigma }_{z}^{aniso}$ *R* ^*2*^ = 0.46, *p* < 0.01; **SBP**: ${F\sigma }_{z}^{iso}$ *R* ^*2*^ = 0.61, *p* < 0.001 and ${F\sigma }_{z}^{aniso}$ **,** *R* ^*2*^ = 0.61, *p* < 0.001.

**Isotropic and anisotropic load bearing fractions in the abdominal aorta**. Circumferential direction: in males, the load bearing fraction of material with isotropic properties ( ${F\sigma }_{\theta }^{iso}$ ) at SBP and DBP correlated positively with age (*R* ^*2*^ = 0.38, *p* < 0.05 and *R* ^*2*^ = 0.36, *p* < 0.05, respectively), while the load bearing fraction of material with anisotropic properties ( ${F\sigma }_{\theta }^{aniso}$ ) correlated negatively with age at SBP and DBP (*R* ^*2*^ = 0.38, *p* < 0.05 and *R* ^*2*^ = 0.36, *p* < 0.05, respectively) (Figure 5A). The fraction of pulsatile isotropic circumferential wall stress correlated positively with age (*R* ^*2*^ = 0.26, *p* = 0.05), while the fraction of pulsatile anisotropic circumferential wall stress correlated negatively with age (*R* ^*2*^ = 0.26, *p* = 0.05). In females, ${F\sigma }_{\theta }^{iso}$ and ${F\sigma }_{\theta }^{aniso}$ did not correlate with age at either SBP or DBP.

Longitudinal direction: Figure 5B shows the load bearing fraction of material with isotropic ( ${F\sigma }_{z}^{iso}$ ) and anisotropic ( ${F\sigma }_{z}^{aniso}$ ) properties at SBP and DBP in males. In males, ${F\sigma }_{z}^{iso}$ at SBP and DBP correlated positively with age (*R* ^*2*^ = 0.61, *p* < 0.001 and *R* ^*2*^ = 0.46, *p* < 0.01, respectively) as well as the fraction of pulsatile isotropic longitudinal wall stress (*R* ^*2*^ = 0.64, *p* < 0.001). Furthermore, ${F\sigma }_{z}^{aniso}$ at SBP and DBP correlated negatively with age (*R* ^*2*^ = 0.61, *p* < 0.001 and *R* ^*2*^ = 0.46, *p* < 0.01, respectively) as well as the fraction of pulsatile anisotropic longitudinal wall stress (*R* ^*2*^ = 0.64, *p* < 0.001). In females, ${F\sigma }_{z}^{iso}$ correlated positively with age at SBP (*R* ^*2*^ = 0.34, *p* < 0.05), while ${F\sigma }_{z}^{aniso}$ correlated negatively with age at DBP (*R* ^*2*^ = 0.34, *p* < 0.05).

### Minor sensitivity test

The computed coefficient of determination (*R* ^*2*^) for baseline was 0.997. In the circumferential direction, a perturbation of 1% in each model parameter, one at a time, caused a change in model parameters: *R*_*0*_ of ca 20.8%, *λ*_*z*_ ca 6.2%, *c* ca 0.11%, *k*_*1*_ ca 0.02%, *k*_*2*_ ca 0.05% and *β* ca 0.48%. In the longitudinal direction, these changes were documented: *R*_*0*_ of ca 21.0%, *λ*_*z*_ ca 42.8%, *c* ca 0.17%, *k*_*1*_ ca 0.04%, *k*_*2*_ ca 0.08% and *β* ca 0.10%. It appears that *R*_*0*_ and *λ*_*z*_ are sensitive to small changes. Inspection of curve forms (not presented) revealed that changing *R*_*0*_ and *λ*_*z*_ caused a parallel translation of the stress curve and did not really affect the slope. Changing *λ*_*z*_ affects longitudinal more than circumferential stress which might be expected. Parameters *c*, *k*_*1*_, *k*_*2*_ and *β* were affected to a smaller extent by a perturbation of 1%.

## Discussion

The main findings in this study are as follows: First, elderly males have higher aortic wall stress and a higher isotropic stress component than elderly females. Second, with age, the load bearing fractions of material with isotropic properties increases while the load bearing fractions of material with anisotropic properties decreases in males but not in females. Since the dominating isotropic and anisotropic constituents are elastin and collagen, respectively (Roach and Burton, 1957), this finding suggests possible changes in the elastin-collagen relationship during remodeling of the AA with respect to both amount and properties. This could, hypothetically, be more extensive in males compared to females.

This study concerns local behavior and therefore the results apply to the AA and might not be valid for other parts of the vascular system. Hence, extrapolations of the results should be made cautiously.

In arterial mechanics, the isotropic property (mainly elastin) dominates at lower blood pressures while the anisotropic property (mainly collagen) involvement becomes gradually larger with increasing pressure (Marsh et al., 2004). Our model is based on a standard Holzapfel-Gasser-Ogden material model that groups all isotropic properties into one single isotropic component, regardless of the origin of isotropy, and similarly for anisotropic properties (Holzapfel et al., 2000; Stalhand, 2009). Hence, isotropic properties from other sources than elastin such as collagen, smooth muscle cells, and all other vascular wall constituents will also be represented by the isotropic component of the model and might change with age. The equivalent holds for anisotropic properties.

The hypothesis of mechanical homeostasis proposes that wall stress affects wall thickness, shear stress affects lumen diameter, and axial force affects longitudinal growth. Increased arterial diameter causes increased wall stress, resulting in outward hypertrophic remodeling of the vascular wall to restore wall stress (Humphrey, 2008). This process is demonstrated in the common carotid artery (Astrand et al., 2005). However, in the male AA, the process appears deficient and wall stress seems to increase with age (Astrand et al., 2005). This age-related increase in PWS in the AA was confirmed in our study. Our data also suggest that the higher PWS in males compared to females, caused by a larger aortic diameter with an insufficient wall thickening, may relate to a higher isotropic component of the PWS in males compared to females. This sex difference in the isotropic component may have developed because of inadequate wall stress regulation in males compared to the preserved age-related remodeling of the AA in females. (Figures 4, 5) (Astrand et al., 2005).

The collagen-to-elastin ratio in the aortic wall is decreased by sex hormones, primarily *via* the inhibition of collagen deposition (Natoli et al., 2005). Furthermore, testosterone has been shown to increase matrix metalloproteinase-3 activity in the aortic wall, causing the degradation of elastin and fibrillin-1, further supporting the concept that the female sex is protected from elastolysis (Natoli et al., 2005). The arterial wall stress-mediated increase in dispersed collagen in the intimal subendothelial layers (Glagov et al., 1993), seems to compensate for lost elastin properties (see above), which in females possibly could contribute to maintaining an arterial wall with isotropic properties without affecting the isotropic stress component.

The load bearing fraction of material with isotropic properties increased with increasing age and the load bearing fraction of material with anisotropic properties decreased at PWS for males (Figure 5). Assuming elastin and collagen have purely isotropic and anisotropic properties, respectively, this presumption could paradoxically indicate that the collagen-to-elastin ratio in that AA wall decreases with age. In contrast, an age-related decrease in concentration but preserved content of elastin and an increase in content and concentration of collagen, have been reported for the AA wall (Tsamis et al., 2013).

Not only the amount of material affects wall stress, but also the stiffness of the material may have an effect. Structural changes, such as damage to lamellar units due to thinning, splitting, and fraying, may affect elastin, whereas collagen displays an increase in the random distribution of the layers of the arterial wall and within specific layers. Moreover, the subendothelial layer of the intima seems to grow thicker from dispersed collagen as a consequence of age-related increases in the vessel radius, and collagen fibers appear to be oriented in a more circumferential direction in the media due to the stretching of the network of helices. Chemical alterations such as elastin glycation and collagen fiber cross-linking also develop with increasing age. These combined structural and chemical changes may indicate two things regarding the material properties of collagen: i) collagen could potentially contribute less to anisotropic material properties in favor of isotropic properties and ii) the age-related changes in collagen might have a disadvantageous consequence on the effect of collagen stiffness (Figures 4, 5) (Glagov et al., 1993; Astrand et al., 2011; Tsamis et al., 2013). Furthermore, a loss of anisotropy might induce remodeling of the vascular wall to preserve structural integrity and manage wall stress at high blood pressures, e.g., SBP. One compensatory remodeling mechanism could be thickening of the vessel wall which has been reported as an age-related phenomenon (Astrand et al., 2005).

Based on our results, we suggest that an age-related material property migration occurs where arterial wall constituents contributing to anisotropic material properties are transformed into constituents with isotropic properties and at the same time an age-related effect on constituent stiffness (Figures 4, 5). This may explain why age-related remodeling in males is insufficient to restore mechanical homeostasis.

The mechanical model combined with the parameter identification algorithm has been validated against a finite element model of an artery with good agreement (Gade et al., 2019). It has also been used to estimate material parameters for aortic vessel wall stiffness (Astrand et al., 2011; Karlsson et al., 2022). Nevertheless, we acknowledge that several limitations can affect the use of PIMMP.

The minor sensitivity test conducted revealed that parameters *R*_*0*_ and *λ*_*z*_ had the largest effect on the *R* ^*2*^ value over a wide range, 6%–40%, when looking at both circumferential and longitudinal directions. Measurement in the longitudinal direction was affected the most when *λ*_*z*_ was perturbed. It reflects a translation of the stress curve and not a change in slope. The rest of the model parameters changed *R* ^*2*^ less than 1%. This is expected since it is known that the parameter identification process is sensitive to the shape of the pressure and radius curves. Furthermore, *R*_*0*_ and *λ*_*z*_ are squared in the invariant *I*, which in turn is squared in Eq. 3; consequently, a small change in *R*_*0*_ and *λ*_*z*_ will have a large impact.

Assessing *R* ^*2*^ value for each participant show a good agreement between Laplace’s law and constitutive stress with values ca 0.98. This supports previous results showing good agreement (Gade et al., 2019).

The mechanical model is based on an assumption of the cylindrical wall being a membrane, i.e., wall thickness should be negligible when compared with the radius. Since the wall thickness-to-radius ratio is ∼0.1–0.2 for the abdominal aorta, the assumption might not be valid (Astrand et al., 2005). Furthermore, a correct model should take into account that the aortic wall consists of three distinct layers which all have different mechanical properties (Holzapfel et al., 2005). However, modeling such a high resolution might introduce dependencies among the parameters during parameter identification (Stalhand et al., 2004). Comparing the membrane model with a three-layer model, the parameters from the membrane model can be regarded as averages, describing the global response of the three layers. Hence, to study age and sex-related phenomena in the aorta, the proposed parameters are adequate.

Using cross-sectional area from formulas based on age (Astrand et al., 2005), may introduce some error. However, since the current data set lacked the needed information for a more precise calculation, an adaptation to the current data set was needed and cross-sectional area as a function of age was assessed to be good enough.

We calculate wall thickness as a function of intima-media thickness. In ultrasound imaging, measuring wall thickness including the adventitia is much more difficult than measuring the intima-media thickness and such measurements are affected by larger measurement variability. Thus, experimental confirmation of the results of modelling is much more difficult to obtain when including the adventitia.

The arbitrary division of a strain-energy function in an isotropic and anisotropic part as suggested by Holzpfel et al. (Holzapfel et al., 2000), may not contribute to increased knowledge from a mechanical point of view since the main purpose of the parts is to aid in the description of the overall wall stress. However, such a division is supported by the work of Roach and Burton (Roach and Burton, 1957) who presented laboratory acquired stress-strain data from enzymatically altered arteries. Therefore, analysis of the isotropic and anisotropic part is motivated since it might offer medical insights to remodeling, material behavior and biological mechanisms. Consequently, the strain-energy function used in this study (Eq. 3) compared to the function used by Schulze-Bauer and Holzapfel (Schulze-Bauer and Holzapfel, 2003), separates the isotropic and anisotropic materials to better account for the structure of the true soft tissue. Furthermore, our strain-energy function allows for a better fit to young subjects, particularly in the low-pressure region where the collagen recruitment is small, and the mechanical behavior is primarily determined by the isotropic material.

It is important to choose a model that suits your needs. In a potential comparison of a four-fiber model with HGO, differences in the results are expected. A four-fiber model could potentially offer more information, in terms of a better “resolution”. However, in our case, there are only two measured variables meaning a model with many parameters, such as a four-fiber model, would be exposed to the risk of over parameterization (Stalhand et al., 2004). Based on available models, we found the HGO model to be sufficient to our needs.

In arteries, there is a residual stress present which the used model does not consider (Fung, 1997; Holzapfel et al., 2000). The major role of the residual stress is to redistribute the stress field, making it uniform through the vessel wall (Holzapfel et al., 2000). Introducing residual stress in a membrane model would only result in an upward shift of the stress levels, which is why the transmural variation of the stress and stretch fields is neglected. Consequently, it is assumed that the membrane stresses are of the same magnitude as the stresses in the arterial wall *in situ*. The assumption also seems reasonable since a global stress balance is used to obtain the stress field to which the model is tuned.

Having access to only pressure and diameter as measured data is a challenge. *Ex vivo* biaxial testing offers a plethora of data which is not obtainable *in vivo*. However, by coupling circumferential and longitudinal stress, as described in the methodology section, it is possible to compute both circumferential and longitudinal stress. Assumptions potentially introduce errors in the model which need to be assessed and properly handled. Our model and the parameter identification process has been validated against a finite element model with good results, indicating that the methodology used produces valid results (Gade et al., 2019).

## Conclusions

Modelling based on *in vivo* measurements showed that males have higher AA wall stress with a higher isotropic stress component, compared to females. Furthermore, the load bearing fraction of material with isotropic properties increased, and the load bearing fraction of material with anisotropic properties decreased with age in males but not in females in the circumferential direction, suggesting vessel wall constituent alterations affecting isotropy and anisotropy as well as the effect of constituent stiffness. The arterial wall model promotes a better understanding of collagen-elastin interactions in the remodeling of vessels during healthy ageing as well as during pathological remodeling when vascular disease is present.

## Data Availability

The raw data supporting the conclusions of this article will be made available by the authors, without undue reservation.

## Abbreviations

AA, abdominal aorta; AAA, abdominal aortic aneurysm; ANOVA, analysis of variance; D, diameter; ∆D , pulsatile diameter: ∆D=D@SBP–D@DBP; DBP, diastolic blood pressure; MAP, mean arterial pressure: MAP=DBP+PP/3; Pa, pascal unit for pressure; PIMMP, parameter identification method for model parameters: The acronym for the model and identification procedure used in this study; PP, ΔP, pulse pressure: Defined as ΔP = SBP—DBP; PWS, peak wall stress: Wall stress at SBP; SBP, systolic blood pressure; SD, standard deviation; σ , wall stress; $\tau_{w}$ , wall shear stress; $\sigma_{n}^{m}$ , superscript *m* denotes wall stress component, subscript *n* denotes direction: *m* = [tot, iso, aniso] *n* = [θ (circumferential), z (longitudinal)]; $\sigma_{\theta },$ circumferential wall stress; $\sigma_{z}$ , longitudinal wall stress; $\sigma_{r}$ , radial wall stress; $\sigma^{tot}$ , total wall stress: Total stress is used to denote $\sigma^{tot}=\sigma^{iso}+\sigma^{aniso};\;\sigma^{iso}$ , isotropic wall stress component: Part of the total wall stress; $\sigma^{aniso}$ , anisotropic wall stress component: Part of the total wall stress; ${F\sigma }_{n}^{m}$ , fraction of wall stress component *m* and total wall stress, in *n* direction: E.g., ${F\sigma }_{\theta }^{aniso}$ denotes fraction of anisotropic wall stress component and total wall stress in circumferential direction; ∆σ , pulsatile wall stress: Defined as ∆σ=σ@SBP−σ@DBP .
